# Interaction of Cigarette Smoking and Polygenic Risk Score on Reduced Lung Function

**DOI:** 10.1001/jamanetworkopen.2021.39525

**Published:** 2021-12-16

**Authors:** Woori Kim, Matthew Moll, Dandi Qiao, Brian D. Hobbs, Nick Shrine, Phuwanat Sakornsakolpat, Martin D. Tobin, Frank Dudbridge, Louise V. Wain, Christine Ladd-Acosta, Nilanjan Chatterjee, Edwin K. Silverman, Michael H. Cho, Terri H. Beaty

**Affiliations:** 1 Systems Biology and Computer Science Program, Ann Romney Center for Neurological Diseases, Department of Neurology, Brigham and Women’s Hospital, Boston, Massachusetts; 2 Harvard Medical School, Boston, Massachusetts; 3 Broad Institute of Harvard and Massachusetts Institute of Technology, Cambridge, Massachusetts; 4 Channing Division of Network Medicine, Department of Medicine, Brigham and Women’s Hospital, Boston, Massachusetts; 5 Division of Pulmonary and Critical Care Medicine, Department of Medicine, Brigham and Women’s Hospital, Boston, Massachusetts; 6 Department of Health Sciences, University of Leicester, Leicester, United Kingdom; 7 Department of Medicine, Faculty of Medicine Siriraj Hospital, Mahidol University, Bangkok, Thailand; 8 National Institute for Health Research Leicester Respiratory Biomedical Research Centre, Glenfield Hospital, Leicester, United Kingdom; 9 Wendy Klag Center for Autism and Developmental Disabilities, Johns Hopkins Bloomberg School of Public Health, Baltimore, Maryland; 10 Department of Epidemiology, Johns Hopkins Bloomberg School of Public Health, Baltimore, Maryland; 11 Department of Biostatistics, Bloomberg School of Public Health, Johns Hopkins University, Baltimore, Maryland; 12 Department of Oncology, School of Medicine, Johns Hopkins University, Baltimore, Maryland; 13 Division of Cancer Epidemiology and Genetics, National Cancer Institute, National Institutes of Health, Department of Health and Human Services, Bethesda, Maryland

## Abstract

**Importance:**

The risk of airflow limitation and chronic obstructive pulmonary disease (COPD) is influenced by combinations of cigarette smoking and genetic susceptibility, yet it remains unclear whether gene-by-smoking interactions are associated with quantitative measures of lung function.

**Objective:**

To assess the interaction of cigarette smoking and polygenic risk score in association with reduced lung function.

**Design, Setting, and Participants:**

This UK Biobank cohort study included UK citizens of European ancestry aged 40 to 69 years with genetic and spirometry data passing quality control metrics. Data was analyzed from July 2020 to March 2021.

**Exposures:**

PRS of combined forced expiratory volume in 1 second (FEV_1_) and percent of forced vital capacity exhaled in the first second (FEV1/FVC), self-reported pack-years of smoking, ever- vs never-smoking status, and current- vs former- or never-smoking status.

**Main Outcomes and Measures:**

FEV_1_/FVC was the primary outcome. Models were used to test for interactions with models, including the main effects of PRS, different smoking variables, and their cross-product terms. The association between pack-years of smoking and FEV_1_/FVC were compared for those in the highest vs lowest decile of estimated genetic risk for low lung function.

**Results:**

We included 319 730 individuals, of whom 24 915 (8%) had moderate-to-severe COPD cases, and 44.4% were men. Participants had a mean (SD) age 56.5 of (8.02) years. The PRS and pack-years were significantly associated with lower FEV_1_/FVC (PRS: β, −0.03; 95% CI, −0.031 to −0.03; pack-years: β, −0.0064; 95% CI, −0.0064 to −0.0063) and the interaction term (β, −0.0028; 95% CI, −0.0029 to −0.0026). A stepwise increment in estimated effect sizes for these interaction terms was observed per 10 pack-years of smoking exposure. The interaction of PRS with 11 to 20, 31 to 40, and more than 50 pack-years categories were β (interaction) −0.0038 (95% CI, −0.0046 to −0.0031); −0.013 (95% CI, −0.014 to −0.012); and −0.017 (95% CI, −0.019 to −0.016), respectively. There was evidence of significant interaction between PRS with ever- or never- smoking status (β, interaction; −0.0064; 95% CI, −0.0068 to −0.0060) and current or not-current smoking (β, interaction; −0.0091; 95% CI, −0.0097 to −0.0084). For any given level of pack-years of smoking exposure, FEV_1_/FVC was significantly lower for individuals in the tenth decile (ie, highest risk) than the first decile (ie, lowest risk) of genetic risk. For every 20 pack-years of smoking, those in the tenth decile compared with the first decile of genetic risk showed nearly a 2-fold reduction in FEV_1_/FVC.

**Conclusions and Relevance:**

COPD is characterized by diminished lung function, and our analyses suggest there is substantial interaction between genome-wide PRS and smoking exposures. While smoking was associated with decreased lung function across all genetic risk categories, the associations were strongest in individuals with higher estimated genetic risk.

## Key Points

**Question:**

Does cigarette smoking interact with genetic risk on the percent of forced vital capacity exhaled in the first second (FEV_1_/FVC)?

**Findings:**

In this UK Biobank cohort study of 319 730 UK citizens, FEV_1_/FVC was associated with polygenic risk score-by-smoking interactions, and smoking was detrimental across all categories of estimated genetic risk, although it was worse for those with the highest estimated genetic risks. For every reported 20 pack-years of smoking, individuals in the top decile compared with the bottom decile of genetic risk showed nearly twice the reduction in FEV_1_/FVC.

**Meaning:**

These findings suggest that elucidating mechanisms for the interaction between smoking and genetic risk could yield greater insight into the chronic obstructive pulmonary disease pathogenesis.

## Teaser

This cohort study assesses the interaction of cigarette smoking and polygenic risk score in association with reduced lung function among participants in the UK Biobank cohort.

## Introduction

Chronic obstructive pulmonary disease (COPD) is characterized by airflow obstruction, traditionally defined by a low percent of forced vital capacity exhaled in the first second (FEV_1_/FVC), and cigarette smoking is the greatest environmental risk factor.^1,2^ Only a minority of smokers develop COPD,^3,4^ and genetic factors are thought to account for some of this variation in susceptibility, with approximately 40% of the variability in spirometric measures of pulmonary function attributed to genetic variation.^5,6,7^ Therefore, it has long been thought that airflow obstruction may develop partially as the result of gene-by-smoking interactions.

Despite the important contribution of both smoking and genetic factors to lung function, compelling evidence for gene-by-smoking interactions has been limited. Genome-wide interaction studies have identified a handful of spirometric- and COPD-associated loci that appear to interact with smoking status,^8,9,10,11,12,13,14^ suggesting at least a portion of the variability in spirometric measures of lung function may be attributable to gene-by-smoking interactions. A major challenge of identifying gene-by-smoking interactions on lung function and risk to COPD is that individual genetic variants tend to be of small effect size and account for a low degree of phenotypic variability in lung function, diminishing the power to detect gene-by-smoking interactions.

Pooling individual genome-wide association studies (GWAS) variants into a single genetic risk score can account for a greater proportion of phenotypic variability,^15,16,17,18,19,20^ and should improve power to detect interactions. Genetic risk scores have been used to investigate gene-by-environment interactions in psychiatric^21^ and cardiovascular diseases.^22^ Aschard et al^23^ were unable to detect individual single nucleotide variation (SNV, formerly single-nucleotide polymorphism [SNP])-by-smoking interactions for FEV_1_/FVC for 26 variants identified as significant in a genome-wide joint meta-analysis of SNV-by-smoking associations of pulmonary function^14^; however, when the authors summed these variants to create a genetic risk score, they found evidence of interaction between the genetic risk score and ever-smoking status.^23^ By contrast, Shrine et al^19^ performed the largest GWAS of lung function to date, developed a genetic risk score including estimated effects of 279 variants showing significant effects on lung function, and reported no evidence of interaction between this genetic risk score and ever-smoking status, although the authors did observe an interaction of the genetic risk score with ever- smoking status on moderate-to-severe COPD. We used a polygenic risk score (PRS) based on GWASs of FEV_1_ and FEV_1_/FVC,^19^ previously constructed for COPD that explained more of the variability in lung function than seen with the 279-variant risk score used by Shrine et al^19^ (approximately 30% vs less than 10%).^20^

We hypothesized that multiple measures of smoking exposure would significantly interact with this genome-wide PRS on FEV_1_/FVC (ie, because it is associated with lower lung function) in the UK Biobank population-based cohort. We chose FEV_1_/FVC as the primary outcome, as it is a measure used to define COPD according to the Global Initiative for Chronic Lung Disease (GOLD) criteria, and the ratio as a continuous measure, is inversely associated with COPD-related events.^2^

## Methods

This cohort study followed Strengthening the Reporting of Observational Studies in Epidemiology (STROBE) reporting guidelines. All participants provided written informed consent, and study protocols were approved by North West Multi-centre Research Ethics Committee and ethical procedures were controlled by the UK Biobank Ethics Advisory Committee.

### Study Population

We included participants from the UK Biobank, a cohort recruiting more than 500 000 individuals from the UK aged 40 to 69 years from 2006 to 2010.^24^ Participants were excluded if spirometry or genetic data did not meet quality control standards; further details on the impact of these inclusion and exclusion criteria are shown in eFigure 1 in the Supplement. Quality control of spirometric data has been previously described.^18,19,24^ Briefly, to determine lung function, FEV_1_ and FVC were derived from the spirometry volume-time series data at the time of study enrollment, as previously reported.^19^

Genotyping was performed as previously described,^19^ using Axiom UK Biobank Lung Exome Variant Evaluation array and Axiom Biobank array (Affymetrix) and imputed to the Haplotype Reference Consortium version 1.1 panel (accepting imputation accuracy *r^2^* > 0.5). We dropped variants with minor allele frequency < 0.01 and those showing deviation from Hardy-Weinberg equilibrium (*P* < 1×10^-6^). We used only participants of European ancestry based on a combination of self-reported ethnicity and k-means clustering of principal components of genetic ancestry, as previously reported.^19^

### Overview of Study Design

The primary outcome was the FEV_1_/FVC ratio, as clinical COPD is characterized by airflow obstruction (FEV_1_/FVC < 0.7), and severity graded based on decrements in FEV_1%_ predicted.^1,2^ We first assessed whether 3 measures of smoking exposure interacted with PRS on quantitative measures of FEV_1_/FVC. We then considered the joint associations of smoking exposures and being in the highest (tenth) decile vs lowest (first) decile of the PRS (ie, highest vs lowest categories of estimated genetic risk). We examined norms of reaction for the association between pack-years of smoking and FEV_1_/FVC for those in the highest (tenth) decile compared with the lowest (first) decile and middle (fifth) decile of estimated genetic risk.

### Smoking Exposures

We examined 3 measures of cigarette smoking exposure: pack-years of smoking, ever- vs never-smoking status, and current smoker vs former- and never-smoking status. All smoking information was obtained by self-report. Pack-years of smoking was examined as continuous and categorical (ie, pack-year categories: ≤10, 10.1-20, 20.1-30, 30.1-40, 40.1-50 and >50; where the reference group is ≤10 pack-years) variables. The category ever-smokers included individuals reporting current smoking, smoking most days, smoking occasionally, or former smoking. Never smokers included those who smoked less than 100 cigarettes in their lifetime. Current smokers included those who reported current smoking, and former smokers included noncurrent smokers who smoked 100 or more cigarettes in their lifetime.

### Polygenic Risk Score for Lung Function

A polygenic risk score (PRS) for lung function was calculated as previously described (eMethods in the Supplement).^20^ Briefly, this PRS was based on GWAS results for FEV_1_ and FEV_1_/FVC in UK Biobank and SpiroMeta,^19^ and was developed using a penalized regression framework accounting for linkage disequilibrium.^25^ PRSs were calculated for FEV_1_ and FEV_1_/FVC and then summed into a composite PRS, which was scaled and centered. The PRS was oriented such that a higher PRS was associated with lower FEV_1_ and FEV_1_/FVC.

### Statistical Analyses

All analyses were done in R version 4.0.3 (R Project for Statistical Computing). The normality of continuous variables was assessed by visual inspection of histograms. Results are reported as mean (SD) or median (IQR), as appropriate. Differences in continuous variables were assessed with *t* tests or Wilcoxon tests, and categorical variables were compared by analysis of variance or Kruskal-Wallis tests, as appropriate. We used α = .05 as a priori level of statistical significance. All hypothesis tests were 2-sided, and data were analyzed from July 2020 to March 2021.

#### Interaction Analyses

We performed multivariable linear regressions of FEV_1_/FVC on the main associations of the combined PRS, smoking exposure, and cross-product interaction terms. We included covariates age, age × age, sex, height, genotyping array, and the first 10 principal components of genetic ancestry in the linear regression model. Age was scaled and centered before squaring. We also performed stratified analyses among those in the lowest and highest deciles of the PRS, separately for never- and ever-smokers.

Investigation of gene-by-environment interactions has been considered to be a deviation from either an additive or multiplicative model. Therefore, we additionally examined the joint effects of smoking and PRS to assess a departure of the observed joint effect from the expected effect under an additive model. We focused on comparing the first (lowest risk) decile and tenth (highest risk) decile of PRS as previously done.^20^ We created a categorical variable with mutually exclusive strata formed by the cross-classification of smoking and PRS (tenth decile vs first decile). The reference category was the group with the lowest relative smoking exposure (eg, pack-years ≤ 10) in the first PRS decile. We then constructed multivariable linear regression models to evaluate the effects of this categorical variable on FEV_1_/FVC, adjusting for age, age × age, sex, height, genotyping array, and the first 10 principal components of genetic ancestry. The expected association for those in the highest decile with the highest smoking exposure was estimated under an additive model and calculated by summing the estimated effect size for the lowest decile vs the highest smoking exposure group and the highest decile vs the lowest smoking exposure group.

#### Norms of Reaction

A norm of reaction describes the association between a phenotype and environmental exposure for a given genotype.^26^ We assessed norms of reaction for pack-years of smoking and FEV_1_/FVC for those in the lowest (first) decile, middle (fifth) decile, and highest (tenth) decile of estimated genetic risk. We plotted pack-years of smoking vs FEV_1_/FVC, stratifying by lowest, middle, and highest deciles of genetic risk. We then compared the slopes of the lowest and highest deciles of genetic risk lines with an analysis of covariance (ANCOVA) using the rstatix R package (R Project for Statistical Computing).^27^ For clinical interpretability, we trained multivariable linear regression models to assess the association of 20 pack-years of smoking with FEV_1_/FVC for those in the highest, middle, and lowest deciles of estimated genetic risk, adjusting for the covariates detailed as previously stated.

As sensitivity analyses, we repeated these analyses in ever-smokers and in a data set excluding all related individuals; to select unrelated individuals, we removed at least 1 individual from each related pair with a kinship coefficient greater than 0.0625, favoring the inclusion of COPD cases. We also transformed reported pack-years of smoking (ie, log, scaling and centering, and rank normalization) and measures of PRS (adding a quadratic term, ie, PRS^2^) to ensure that the effects of interaction terms were not because of misspecification of the main effects of smoking or PRS. To ensure the robustness of our results to the normality of the outcome, we repeated our analyses after log-transforming FEV_1_/FVC.

## Results

### Characteristics of Study Participants

We included 319 730 participants; 24 915 participants met criteria for moderate-to-severe COPD cases (GOLD spirometry grades 2 to 4)^1^; 38 713 had preserved ratio with impaired spirometry (PRISm);^28^ and 256 102 met criteria for GOLD spirometry grades 0 and 1. Participants had a mean (SD) age of 56.5 (8.02), and 141 864 (44.4%) were male. Characteristics of study participants are shown in Table 1.

**Table 1.** Characteristics of Study Participants

Characteristics	Overall, No. (%)
No.	319 730
Age, mean (SD)	56.45 (8.02)
Sex	
Female	177 866 (55.6)
Male	141 864 (44.4)
Pack-years of smoking, median (IQR)	0 (0-11.00)
Smoking status	
Former/never	287 445 (89.9)
Current	32 242 (10.1)
Ever^a^	146 679 (45.9)
FEV_1_% predicted, mean (SD)	92.17 (16.01)
FEV_1_/FVC, mean (SD)	0.76 (0.06)
Decile of polygenic risk score	
Lowest	31 973 (10.0)
Highest	31 973 (10.0)

Abbreviations: FEV_1_, forced expiratory volume in 1 second; FVC, forced vital capacity.

^a^ 43 individuals were ever-smokers and did not provide any details regarding current or former smoking.

### Interaction of a Polygenic Risk Score With Smoking

**Figure 1.**
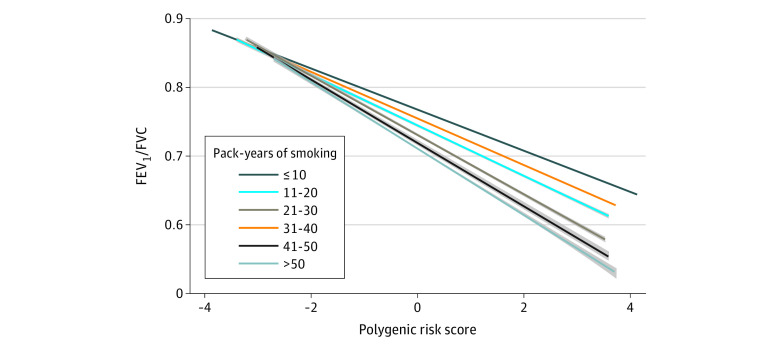
The Relationship Between Polygenic Risk Score and FEV_1_/FVC by Pack-Years of Smoking FEV_1_ indicates forced expiratory volume in 1 second; FVC, forced vital capacity. The shading indicates 95% CI.

The PRS was weakly correlated with pack-years of smoking (r = 0.041; *P* < .001) (eFigure 2 in the Supplement). The association between PRS and FEV_1_/FVC stratified by pack-years of smoking categories is illustrated in Figure 1. The PRS was associated with lower FEV1/FVC across all pack-years of smoking categories, and the magnitude of the association of PRS on reduced FEV1/FVC increased with higher pack-years of smoking. In multivariable analyses, PRS (β = −0.0304; 95% CI, −0.0307 to −0.0302) and pack-years of smoking categories were associated with FEV_1_/FVC (*P* < .001), and estimated effect sizes increased with each incremental category of smoking exposure (Table 2). The same incremental trend for interaction terms between PRS and pack-years of smoking categories was observed (all tests for interaction yielded *P* < .001). The PRS and pack-years were significantly associated with lower FEV_1_/FVC (PRS: β, −0.03; 95% CI, −0.031 to −0.03; pack-years: β, −0.0064; 95% CI, −0.0064 to −0.0063). Considering pack-years of smoking as a continuous variable (eTable 1 in the Supplement), the cross-product interaction term was also associated with FEV_1_/FVC (β [interaction] = −0.0028; 95% CI, −0.0029 to −0.0026; *P* < .001). We also performed transformations of pack-years of smoking, PRS, or FEV_1_/FVC, and the PRS × pack-years interaction term was significant in each analysis (all *P* < .001) (eTable 2 to eTable 4 in the Supplement).

**Table 2.** Regression of FEV_1_/FVC on Main Associations of PRS and Pack-Years of Smoking and Their Cross-Product Term, Adjusting for Covariates^a^

Variable	β (95% CI)	*P* value
PRS	−0.03 (−0.031 to −0.03)	<.001
Pack-years		
11-20	−0.011 (−0.012 to −0.01)	<.001
21-30	−0.014 (−0.015 to −0.014)	<.001
31-40	−0.026 (−0.027 to −0.025)	<.001
41-50	−0.034 (−0.035 to −0.033)	<.001
>50	−0.04 (−0.041 to −0.038)	<.001
PRS × pack-years category		
PRS × 11-20	−0.0038 (−0.0046 to −0.0031)	<.001
PRS × 21-30	−0.0068 (−0.0077 to −0.006)	<.001
PRS × 31-40	−0.013 (−0.014 to −0.012)	<.001
PRS × 41-50	−0.015 (−0.017 to −0.014)	<.001
PRS × >50	−0.017 (−0.019 to −0.016)	<.001

Abbreviations: FEV_1_, forced expiratory volume in 1 second; FVC, forced vital capacity; PRS, polygenic risk score.

^a^ Covariates include age, age×age, sex, height, genotyping array, and principal components of genetic ancestry. Pack-years of smoking is included as a categorical variable with 10 or more pack-years as the reference group.

The association between the PRS and FEV_1_/FVC stratified by ever-smoking vs never-smoking and current-smoking vs former- or never-smoking statuses are shown in eFigure 3A and eFigure 3B in the Supplement, respectively. Ever-smoking and the PRS × ever-smoking status interaction term were significantly associated with FEV_1_/FVC (both *P* < .001) (eTable 5 in the Supplement). Similarly, current smoking status and the PRS × current-smoking status interaction term were significantly associated with FEV_1_/FVC (both *P* < .001) (eTable 6 in the Supplement). In stratified analyses, we observed similar results between PRS and smoking exposures (eTable 7 and eFigure 4 in the Supplement). Additionally, ever-smoking status and the PRS × ever-smoking status interaction term were significantly associated with FEV_1_/FVC in the lowest (β [interaction] = −.0033; 95% CI, −0.0058 to −0.00085; *P* = .0082) and highest (β [interaction] = −.0095; 95% CI, −0.013 to −0.0056; *P* < .001) deciles of estimated genetic risk (eTable 7 in the Supplement).

**Figure 2.**
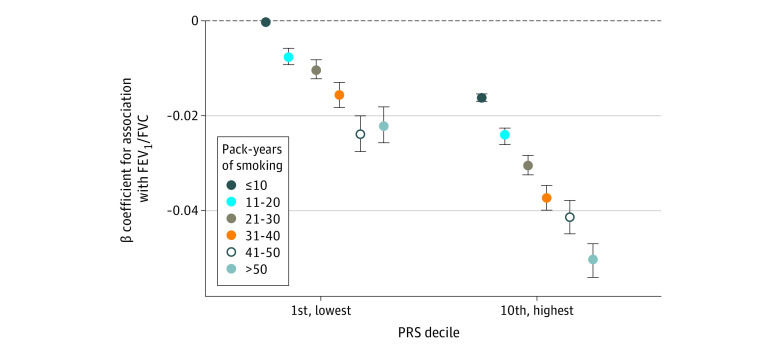
Joint Associations of Pack-Years of Smoking Category and PRS Decile PRS indicates polygenic risk score. The error bars indicate 95% CI.

Being in the lowest decile of estimated genetic risk and having more than 50 pack-years of smoking exposure (β = −.022; 95% CI, −0.026 to −0.018; *P* < .001) had a similar estimated effect size as being in the highest decile of genetic risk and having 11 to 20 pack-years of smoking exposure (β = −0.024; 95% CI, −0.026 to −0.023; *P* < .001). The joint effects of pack-years of smoking categories and PRS are shown in Figure 2. We observed a greater effect size of being in the highest decile of genetic risk and having more than 50 pack-years of smoking exposure (β = −0.051; 95% CI, −0.054 to −0.047) than would be expected, confirming possible interaction between the PRS and pack-years of smoking. We observed a similar association for those in the highest genetic risk decile who were current smokers (eTable 8 in the Supplement), but a nonsignificant difference for ever-smokers in the highest genetic risk decile (eTable 9 in the Supplement).

### Norms of Reaction for Highest vs Lowest Estimated Genetic Risk Deciles

**Figure 3.**
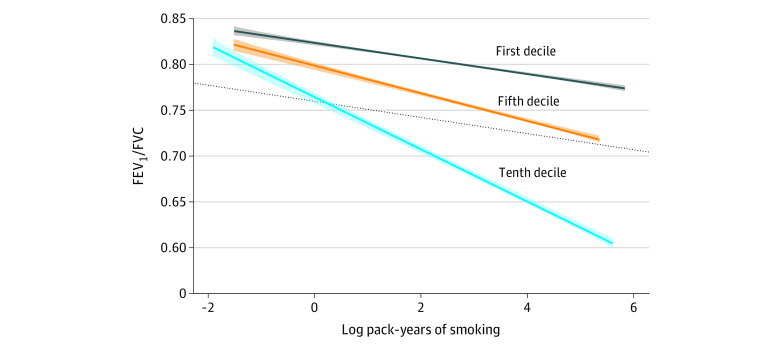
Norms of Reaction for Those With High, Middle, and Low Estimated Genetic Risk for COPD The slopes for the first and tenth decile were significantly different in the analysis of covariance (*P* < .001). The dotted line indicates the absence of the interaction between PRS decile and pack-years of smoking on FEV_1_/FVC. The first, fifth, and tenth decile indicate lowest, middle, and highest decile, respectively. COPD indicates chronic obstructive pulmonary disease; FEV_1_/FVC, percent of forced vital capacity exhaled in the first second, PRS, polygenic risk score. The shading indicates 95% CI.

In Figure 3, we show different norms of reaction for the associations of pack-years of smoking on FEV_1_/FVC among those in the highest (tenth), middle (fifth), and lowest (first) deciles of estimated genetic risk. For any given level of pack-years of smoking, those in the highest PRS decile had lower FEV_1_/FVC compared with those in the lowest decile of PRS. Analysis of covariance confirmed that the slopes of the lines for the highest and lowest decile of PRS are significantly different (*P* < .001) (Figure 3). We observed similar results in ever-smokers (eFigure 5 in the Supplement). For every 20 pack-years of smoking, those in the first (ie, lowest risk) decile had a change of β = −0.0084 (95% CI, −0.0091 to −0.0076) in FEV_1_/FVC, while those in the tenth (ie, highest risk) decile of estimated genetic risk had a change of β = −0.017 (95% CI, −0.019 to −0.016), representing an approximately 2-fold reduction in FEV_1_/FVC for every 20 pack-years of smoking for those in the highest compared with the lowest decile of estimated genetic risk.

## Discussion

In this study of more than 300 000 UK Biobank participants, we found that 3 measures of smoking exposure interacted with PRS on the quantitative measure of lung function (FEV_1_/FVC). As expected, smoking was detrimental to lung function across all categories of estimated genetic risk. However, for any given level of pack-years of smoking exposure, those at the highest genetic risk showed lower FEV_1_/FVC than those with the lowest estimated genetic risk. Furthermore, the outcomes associated with heavy smoking and being in the highest decile of estimated genetic risk were greater than would be expected based on the additive effects of both risk factors. These results support the idea that diminished pulmonary function (ie, a measure of airflow obstruction) is, at least partially, due to gene-by-smoking interactions, and those in higher genetic risk categories are more susceptible to the deleterious effects of smoking.

Compared with previous studies, our study included more participants, leveraged a more effective measure of the genetic predisposition for low lung function (ie, PRS), examined 3 different measures of smoking exposure (ie, pack-years, ever-smoking, current smoking), and examined norms of reactions for those in the highest decile compared with the lowest decile of estimated genetic risk. Our findings are consistent with Aschard et al^23^ who reported an interaction between ever-smoking status and a genetic risk score for FEV_1_/FVC based on 26 different variants. In a family-based study of the rs28929474 variant (Z allele) in *SERPINA1*, which leads to alpha-1 antitrypsin deficiency (AATD) and greatly increased risk of emphysema, there was a significant genotype-by-smoking interaction on FEV_1_.^29^ A strong smoking interaction of rs28929474 heterozygote status with ever-smoking on lung function and COPD has been reported in UK Biobank.^30^ GWAS have confirmed the interaction of smoking with rs28927474 on risk to COPD,^11^ and identified gene-by-smoking interactions at several other loci.^8,9,10,11,12,13,14^ Recently, a study of incident COPD found evidence for a gene-by-smoking interaction, further supporting our findings and suggesting that the relationship of the PRS to other related phenotypes or outcomes may also interact with smoking or other measures.^31^ In addition, our study quantifies this interaction with respect to lung function rather than COPD status, provides finer resolution regarding level of smoking exposure and genetic interactions, demonstrates robust interactions despite a range of model specifications, and provides a clinical framework for considering estimated genetic risk and susceptibility to the damaging effects of cigarette smoke (ie, the association between 20 pack-years of smoking and FEV1/FVC reduction).

By contrast, Shrine et al^19^ constructed a genetic risk score from 279 variants associated with lung function but did not observe any evidence of interaction with ever-smoking status on FEV_1_/FVC; further, the authors reported an interaction with moderate-to-severe COPD status in the opposite direction as expected. A prior GWAS selected approximately 50 000 individuals with low, average, and high FEV_1_ and reported no gene-smoking interactions at genome-wide significance.^32^ The reasons for disparate findings between these studies and the current study are unclear but may represent the effects of different loci with varying degrees of the interaction effect.^4,19,20,33^ While the PRS used in the current study was derived from the Shrine et al^19^ GWAS results, not all variants reached genome-wide significance, and consequently included many more variants (approximately 2.5 M); this more refined level of estimated genetic risk may have provided the power to detect gene-by-smoking interactions. The particular PRS used in these analyses likely influences the findings of studies evaluating gene-by-smoking interactions.

Smoking was associated with poor outcomes even to those with low estimated genetic risk, and the effects were greater for those with high estimated genetic risk. For any given level of pack-years of smoking, those in the highest decile had lower FEV_1_/FVC compared with those in the lowest decile of estimated genetic risk. These findings are in contrast to observations in cardiovascular disease, where the association between smoking and coronary heart disease was greater for those in the lowest compared with the highest tertile of a PRS.^22^ This difference may reflect that many individuals can develop coronary disease in the absence of cigarette smoking and that smoking is a greater risk factor for those with low polygenic risk for coronary disease. Meanwhile, airflow obstruction primarily occurs in the setting of cigarette smoking exposure. Current smokers with high estimated genetic risk demonstrated a greater reduction in lung function than expected, but this association was not observed in individuals who had ever smoked, suggesting gene-by-smoking interactions may be dose-dependent and that current smoking has a direct effect on lung function. Furthermore, those with low estimated genetic risk and high smoking exposure had similar risk for low FEV_1_/FVC as those with high genetic risk and low smoking exposure. Taken together, these results emphasize that abstaining from smoking is crucial to preventing obstructive lung disease regardless of an individual’s estimated genetic risk and that those in the highest risk groups might benefit from intensive smoking cessation measures with respect to the phenotypes examined in this study.

The PRS used in our study was based on a prior GWAS of lung function.^19^ In this GWAS, along with a GWAS of COPD status, significant variants are involved in the pathways related to lung growth, as well as elastic fiber and extracelluar matrix, ciliogenesis, and transforming growth factor-β.^18,19^ We have previously shown PRS is associated with lung structure, such as emphysema and airway measures, as well as reduced lung function growth patterns that can lead to spirometric COPD in early adulthood.^20,33^ Thus, the interactions with this PRS could reflect interaction with a number of different biologic processes influenced by these genetic variants. Our results suggest that the PRS includes variants that represent biological pathways by which smoking exerts deleterious effects. Some of these variants may act to confer resilience^34,35^ or susceptibility to the effects of cigarette smoke. Further research to elucidate the potential contributions of key variants used in the PRS and their biological mechanisms underlying the interaction between genetics and smoking on lung function is needed, which could be facilitated by functional studies, examination of other related phenotypes, and multiomics follow up studies. For example, the effect of occupational exposures was modified by rs9931086 in *SLC38A8* on FEV_1_, and network analyses suggested inflammatory processes involving CTLA-4, HDAC, and PPAR-α, may provide mechanistic links for the observed interaction^36^; however, this was a small study that needs replication.

Strengths of this study include use of a large volunteer cohort, using the most powerful measure for genetic risk for low lung function available to date (ie, a genome-wide PRS), and comparing individuals at extremes of estimated genetic risk. Our study finds that cigarette smoking has a detrimental association with lung function across all levels of genetic risk but has a particularly deleterious effect on those at highest genetic risk of reduced lung function. Polygenic risk scores can contribute to the assessment of COPD risk at all levels of smoking exposure. Knowledge of a person’s genetic risk could allow for earlier diagnosis at lower levels of smoking exposure. Our results also provide a framework for identifying those most susceptible to the harmful effects of smoking who could be targeted for individualized and public health smoking cessation or prevention programs. The effectiveness of targeted genetically informed smoking cessation interventions is unclear, although there is evidence that knowledge of genetic risk for AATD can increase smoking cessation.^37^ Finally, further investigation into the biological mechanisms by which high genetic risk groups exhibit greater susceptibility to cigarette smoking exposure may identify targets for personalized therapeutics. Clinical use of the PRS will depend on dissecting biological mechanisms of susceptibility to the harmful effects of smoking.

### Limitations

Limitations, inherent to study design, include that the UK Biobank is a single cohort observed in cross-section. Examining the effects of gene-by-smoking interactions on incident COPD should be pursued. We were not able to model the time-varying effects of smoking exposure. We used self-reported smoking measures, which is prone to recall bias. We considered only smoking exposure to study gene-by-environment interactions on lung function. Besides cigarette smoking, other environment risk factors, such as occupational exposure and air pollution, should be considered in future interaction studies. We could not examine the effects of gene-by-smoking interactions on lung function in early life. A substantial number of COPD cases occur among individuals whose lung function fails to reach optimal levels in early adulthood.^38^ Future studies can be done to investigate whether similar gene-by-smoking interactions occur among those who develop airflow obstruction at an early age. The PRS was partially developed using samples from UK Biobank, leading to overfitting of the PRS with respect to spirometric measures; while this issue should not affect interaction assessments, these results should ideally be replicated in future studies. However, the strong effect sizes and robustness to stratified and transformed analyses do lend confidence to our results. We included European-ancestry participants only because the PRS was derived solely from Europeans. Identification of causal variants and genetic prediction in single ancestry populations demonstrate limited portability to multiancestry populations.^39,40^ Thus, the generalizability of the findings of our study to other populations is not certain. The 279 lung function variants from Shrine et al^19^ was curated to ensure variants for smoking behavior were excluded, but the PRS used in the current study included approximately 2.5 million variants and was not similarly curated. Including variants that are causal for smoking behavior could bias the interaction term.^41^ We observed a very weak correlation between this PRS and smoking exposure in UK Biobank, which might be driven by our large sample size, or since we did not exclude smoking related regions, there could be some smoking-related genetic variants included in the PRS. Previously no correlation with smoking in case-control cohorts was observed,^20^ suggesting that the PRS used in the current study largely reflects the genetics of lung function.

## Conclusions

In conclusion, diminished FEV_1_/FVC and airflow obstruction, which are characteristic of COPD, may be partially attributable to gene-by-smoking interactions. As expected, smoking was harmful across all genetic risk groups but worse for those in the highest decile of estimated genetic risk. Large-scale replication and further investigations into mechanisms of interaction are needed.

## Notes

Supplement.
eMethods.
**eTable 1.** Multivariable linear regression of the form FEV1/FVC ~ PRS + Pack Years of Smoking + PRS×Pack-Years of Smoking + Covariates **eTable 2.** Multivariable Linear Regression of the Form FEV1/FVC ~ PRS + Pack years of smoking + PRS×pack-years of smoking + covariates **eTable 3.** Multivariable Linear Regression of the Form FEV1/FVC ~ PRS + PRS×PRS + Pack Years of Smoking + PRS×Pack-Years of Smoking + Covariates **eTable 4.** Multivariable Linear Regression of the Form log(FEV1/FVC) ~ PRS + Pack Years of Smoking + PRS×Pack-Years of Smoking + Covariates **eTable 5.** Multivariable Regression of the Form FEV1/FVC ~ PRS + Ever-Smoking Status + PRS×Ever- Smoking Status + Covariates **eTable 6.** Multivariable Linear Regression of the Form FEV1/FVC ~ PRS + Current Smoking Status + PRS×Current Smoking Status + Covariates **eTable 7.** Cohort Stratified by Ever- vs Never Smoking Status and Highest vs Lowest PRS Decile **eTable 8.** Joint Association of Current-Smoking Status and Highest vs Lowest Decile of PRS **eTable 9.** Joint Association of Ever-Smoking and Being in the Highest vs Lowest Decile of the PRS **eFigure 1.** Participants Included in the Study **eFigure 2.** Scatterplot of the Polygenic Risk Score and Pack-Years of Smoking Exposure **eFigure 3.** The Relationship Between the Polygenic Risk Score and FEV1/FVC **eFigure 4.** The relationship between PRS and FEV_1_/FVC by Pack Years of Smoking and Current vs Former Smoking Status in Ever-Smokers **eFigure 5.** Norms of Reaction for Those With Highest (Tenth) vs Lowest (First) and Middle (Fifth) Genetic Risk for COPD in Individuals Who Had Ever Smoked
eReferences
