# Supplementary material for: Interaction of Cigarette Smoking and Polygenic Risk Score on Reduced Lung Function
Source: JAMA Netw Open. 2021 Dec 16;4(12):e2139525. doi: 10.1001/jamanetworkopen.2021.39525 (PMC8678715; doi:10.1001/jamanetworkopen.2021.39525)
Supplement: Supplement. — eMethods. eTable 1. Multivariable linear regression of the form FEV1/FVC ~ PRS + Pack Years of Smoking + PRS×Pack-Years of Smoking + Covariates eTable 2. Multivariable Linear Regression of the Form FEV1/FVC ~ PRS + Pack years of smoking + PRS×pack-years of smoking + covariates eTable 3. Multivariable Linear Regression of the Form FEV1/FVC ~ PRS + PRS×PRS + Pack Years of Smoking + PRS×Pack-Years of Smoking + Covariates eTable 4. Multivariable Linear Regression of the Form log(FEV1/FVC) ~ PRS + Pack Years of Smoking + PRS×Pack-Years of Smoking + Covariates eTable 5. Multivariable Regression of the Form FEV1/FVC ~ PRS + Ever-Smoking Status + PRS×Ever- Smoking Status + Covariates eTable 6. Multivariable Linear Regression of the Form FEV1/FVC ~ PRS + Current Smoking Status + PRS×Current Smoking Status + Covariates eTable 7. Cohort Stratified by Ever- vs Never Smoking Status and Highest vs Lowest PRS Decile eTable 8. Joint Association of Current-Smoking Status and Highest vs Lowest Decile of PRS eTable 9. Joint Association of Ever-Smoking and Being in the Highest vs Lowest Decile of the PRS eFigure 1. Participants Included in the Study eFigure 2. Scatterplot of the Polygenic Risk Score and Pack-Years of Smoking Exposure eFigure 3. The Relationship Between the Polygenic Risk Score and FEV1/FVC eFigure 4. The relationship between PRS and FEV1/FVC by Pack Years of Smoking and Current vs Former Smoking Status in Ever-Smokers eFigure 5. Norms of Reaction for Those With Highest (Tenth) vs Lowest (First) and Middle (Fifth) Genetic Risk for COPD in Individuals Who Had Ever Smoked eReferences [file jamanetwopen-e2139525-s001.pdf]

Supplemental Online Content

Kim W, Moll M, Qiao D, et al. Interaction of cigarette smoking and polygenic risk score on reduced lung function. *JAMA Netw Open*. 2021;4(12):e2139525.
doi:10.1001/jamanetworkopen.2021.39525

eMethods
eTable 1. Multivariable linear regression of the form FEV1/FVC ~ PRS + Pack Years of Smoking + PRS×Pack-Years of Smoking + Covariates
eTable 2. Multivariable Linear Regression of the Form FEV1/FVC ~ PRS + Pack years of smoking + PRS×pack-years of smoking + covariates
eTable 3. Multivariable Linear Regression of the Form FEV1/FVC ~ PRS + PRS×PRS + Pack Years of Smoking + PRS×Pack-Years of Smoking + Covariates
eTable 4. Multivariable Linear Regression of the Form log(FEV1/FVC) ~ PRS + Pack Years of Smoking + PRS×Pack-Years of Smoking + Covariates
eTable 5. Multivariable Regression of the Form FEV1/FVC ~ PRS + Ever Smoking Status + PRS×Ever Smoking Status + Covariates
eTable 6. Multivariable Linear Regression of the Form FEV1/FVC ~ PRS + Current Smoking Status + PRS×Current Smoking Status + Covariates
eTable 7. Cohort Stratified by Ever vs Never Smoking Status and Highest vs Lowest PRS Decile
eTable 8. Joint Association of Current-Smoking Status and Highest vs Lowest Decile of PRS
eTable 9. Joint Association of Ever-Smoking and Being in the Highest vs Lowest Decile of the PRS
eFigure 1. Participants Included in the Study
eFigure 2. Scatterplot of the Polygenic Risk Score and Pack-Years of Smoking Exposure
eFigure 3. The Relationship Between the Polygenic Risk Score and FEV1/FVC
eFigure 4. The relationship between PRS and FEV_1/FVC by Pack Years of Smoking and Current vs Former Smoking Status in Ever Smokers
eFigure 5. Norms of Reaction for Those With Highest (Tenth) vs Lowest (First) and Middle (Fifth) Genetic Risk for COPD in Individuals Who Had Ever Smoked
eReferences

This supplemental material has been provided by the authors to give readers additional information about their work.

© 2021 Kim W et al. *JAMA Network Open.*

eMethods

Polygenic risk score for lung function

A polygenic risk score (PRS) for lung function was calculated as previously described [1]. Briefly, we assessed the contribution of genetic variants from GWASs of FEV_1 and FEV_1/FVC in the UK Biobank and SpiroMeta [2]. We applied a penalized regression framework, accounting for linkage disequilibrium (LD) to develop individual PRSs for FEV_1 and FEV_1/FVC separately. We created a single combined PRS using a weighted sum determined from training data as: $PRS_{Combined}=0.43847 \times PRS_{FEV1}+ 0.58833 \times PRS_{FEV1/FVC}$. The combined PRS, which included approximately 2.5 million SNPs, was centered and scaled to have a mean of zero and a standard deviation of 1. This combined score is used in our analysis.

eTable 1. Multivariable linear regression of the form FEV_1/FVC ~ PRS + Pack Years of Smoking + PRS×Pack-Years of Smoking + Covariates

**Variable**	**Beta (95% CI)**	***P***
PRS	-0.03 (-0.031--0.03)	< 0.001
Pack years	-0.0064 (-0.0066--0.0063)	< 0.001
PRS X Pack years	-0.0028 (-0.0029--0.0026)	< 0.001

Covariates include age, age×age, sex, height, genotyping array, principal components of genetic ancestry. Pack years of smoking is included as a continuous variable. PRS = polygenic risk score [1,2].

eTable 2. Multivariable Linear Regression of the Form FEV_1/FVC ~ PRS + Pack years of smoking + PRS×pack-years of smoking + covariates.

**Variable**	**Beta (95% CI)**	**P**
Log-transformed pack years		
PRS	-0.021 (-0.022--0.022)	< 0.001
Pack years	-0.0078 (-0.0083--0.0083)	< 0.001
PRS X Pack years	-0.0058 (-0.0063--0.0063)	< 0.001
Scaled and centered pack years		
PRS	-0.033 (-0.034--0.034)	< 0.001
Pack years	-0.0072 (-0.0076--0.0076)	< 0.001
PRS X Pack years	-0.004 (-0.0044--0.0044)	< 0.001
Rank-normalized pack years		
PRS	-0.027 (-0.028--0.028)	< 0.001
Pack years	-0.016 (-0.017--0.017)	< 0.001
PRS X Pack years	-0.0095 (-0.01--0.01)	< 0.001

Covariates include age, age×age, sex, height, genotyping array, principal components of genetic ancestry. Pack years of smoking were transformed prior to analyses to ensure robustness of results to any misspecification of the main effects of smoking. PRS = polygenic risk score.

eTable 3. Multivariable Linear Regression of the Form FEV1/FVC ~ PRS + PRS×PRS + Pack Years of Smoking + PRS×Pack-Years of Smoking + Covariates

Variable	*Beta (95% CI)*	*P*
Adding quadratic term (Continuous variable of pack-years)		
PRS	-0.03 (-0.031--0.031)	< 0.001
Pack-years of smoking	-0.00064 (-0.00066--0.00066)	< 0.001
PRS squared	-0.0022 (-0.0023--0.0023)	< 0.001
PRS X pack-years of smoking	-0.00027 (-0.00028--0.00028)	< 0.001
Adding quadratic term (Categorical variable of pack-years)		
PRS	-0.03 (-0.031--0.031)	< 0.001
PRS squared	-0.0021 (-0.0023--0.0023)	< 0.001
PRS X 11-20 pack years category	-0.0038 (-0.0046--0.0046)	< 0.001
PRS X 21-30 pack years category	-0.0068 (-0.0076--0.0076)	< 0.001
PRS X 31-40 pack years category	-0.013 (-0.014--0.014)	< 0.001
PRS X 41-50 pack years category	-0.015 (-0.016--0.016)	< 0.001
PRS X >50 pack years category	-0.017 (-0.018--0.018)	< 0.001

Covariates include age, age*age, sex, height, genotyping array, principal components of genetic ancestry. PRS was transformed to ensure the robustness of results to any misspecification of the main effects of PRS. PRS = polygenic risk score.

eTable 4. Multivariable Linear Regression of the Form log(FEV1/FVC) ~ PRS + Pack Years of Smoking + PRS×Pack-Years of Smoking + Covariates

**Variable**	**Beta (95% CI)**	***P***
Continuous variable of pack-years		
PRS	-0.041 (-0.041 to -0.041)	< 0.001
Pack-years of smoking	-0.00091 (-0.00093 to -0.00093)	< 0.001
PRS X pack-years of smoking	-0.00049 (-0.00051 to -0.00051)	< 0.001
Categorical variable of pack-years		
PRS	-0.041 (-0.041 to -0.041)	< 0.001
PRS X 11-20 pack years category	-0.0063 (-0.0074 to -0.0074)	< 0.001
PRS X 21-30 pack years category	-0.011 (-0.013 to -0.013)	< 0.001
PRS X 31-40 pack years category	-0.022 (-0.024 to -0.024)	< 0.001
PRS X 41-50 pack years category	-0.027 (-0.029 to -0.029)	< 0.001
PRS X >50 pack years category	-0.031 (-0.033 to -0.033)	< 0.001

Covariates include age, age×age, sex, height, genotyping array, principal components of genetic ancestry. FEV_1/FVC was transformed to log scale to ensure the robustness of our results to the normality of outcome. PRS indicates polygenic risk score.

eTable 5. Multivariable Regression of the Form FEV_1/FVC ~ PRS + Ever Smoking Status + PRS×Ever Smoking Status + Covariates

**Variable**	**Beta (95% CI)**	***P***
PRS	-0.03 (-0.031--0.03)	< 0.001
Ever smoker	-0.016 (-0.016--0.015)	< 0.001
PRS X Ever smoker	-0.0064 (-0.0068--0.006)	< 0.001

Covariates include age, age×age, sex, height, pack years of cigarette smoking, genotyping array, and principal components of genetic ancestry. Ever-smoking status was compared to never-smoking status. PRS indicates polygenic risk score.

eTable 6. Multivariable Linear Regression of the Form FEV1/FVC ~ PRS + Current Smoking Status + PRS×Current Smoking Status + Covariates

Variable	*Beta (95% CI)*	*P*
PRS	-0.032 (-0.032--0.032)	< 0.001
Current smoker	-0.027 (-0.028--0.027)	< 0.001
PRS X Current smoker	-0.0091 (-0.0097--0.0084)	< 0.001

Covariates include age, age×age, sex, height, pack years of cigarette smoking, genotyping array, and principal components of genetic ancestry. Current smoking status was compared to former-/never smoking status. PRS indicates polygenic risk score.

eTable 7. Cohort Stratified by Ever vs Never Smoking Status and Highest vs Lowest PRS Decile

Stratum	*Variable*	*Beta (95% CI)*	*P*
	Ever smoking	-0.014 (-0.018--0.01)	< 0.001
Lowest PRS decile	PRS	-0.024 (-0.025--0.022)	< 0.001
	PRS X Ever smoking interaction	-0.0033 (-0.0058--0.00085)	0.008
	Ever smoking	-0.011 (-0.017--0.0042)	0.001
Highest PRS decile	PRS	-0.037 (-0.04--0.035)	< 0.001
	PRS X Ever smoking interaction	-0.0095 (-0.013--0.0056)	< 0.001
Never smokers	PRS	-0.03 (-0.03--0.03)	< 0.001
Never smokers	Top decile of PRS	-0.095 (-0.097--0.094)	< 0.001
	Pack years of cigarette smoking	-0.00047 (-0.00049--0.00045)	< 0.001
Ever smokers	PRS	-0.031 (-0.032--0.031)	< 0.001
	PRS X Pack years of smoking interaction	-0.00026 (-0.00028--0.00024)	< 0.001
	Pack years of cigarette smoking	-3.1e-05 (-0.00011-4.6e-05)	0.43
Ever smokers	Top decile of PRS	-0.1 (-0.1--0.099)	< 0.001
	Top decile of PRS X Pack years of smoking interaction	-0.00075 (-0.00084--0.00065)	< 0.001

Interaction terms were evaluated between dichotomized PRS (denoted "Top decile of PRS") or continuous PRS (denoted "PRS") and the appropriate smoking variable. Each box represents a single model. PRS = Polygenic risk score.

eTable 8. Joint Association of Current-Smoking Status and Highest vs Lowest Decile of PRS

	Lowest decile of PRS (beta (95% CI))	*Highest decile of PRS (beta (95% CI))*
Former-/Never-smokers	0 (ref)	-0.017 (-0.017--0.016)
Current smokers	-0.016 (-0.018--0.013)	-0.037 (-0.039--0.035)

PRS = polygenic risk score.

eTable 9. Joint Association of Ever-Smoking and Being in the Highest vs Lowest Decile of the PRS

	Lowest decile of PRS (beta (95% CI))	*Highest decile of PRS (beta (95% CI))*
Never smokers	0 (ref)	-0.016 (-0.017--0.015)
Ever smokers	-0.0026 (-0.0038--0.0013)	-0.022 (-0.023--0.02)

PRS = polygenic risk score.

eFigure 1. Participants Included in the Study.

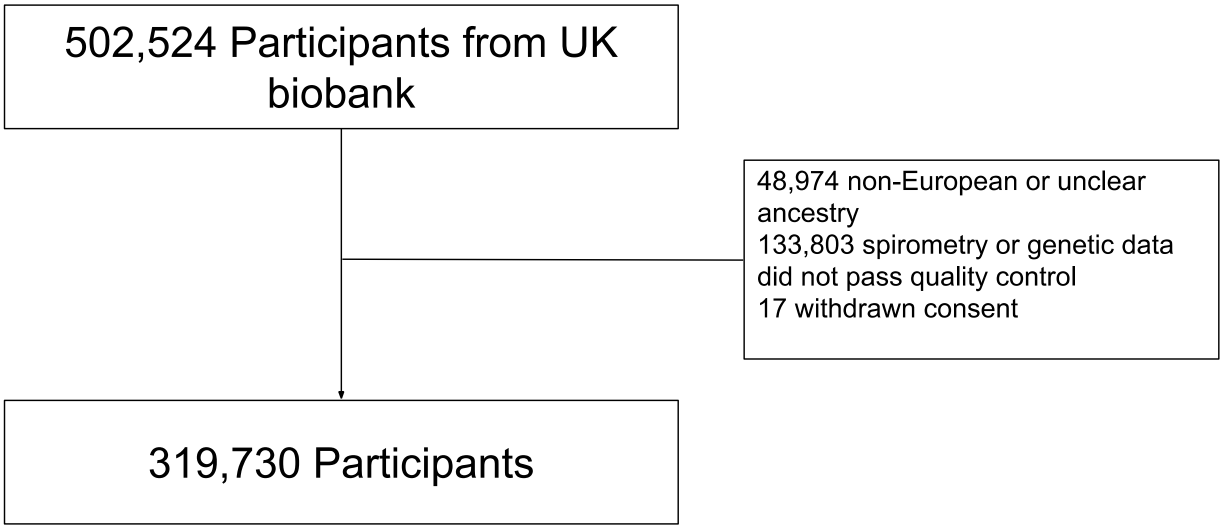

eFigure 2. Scatterplot of the Polygenic Risk Score and Pack-Years of Smoking Exposure

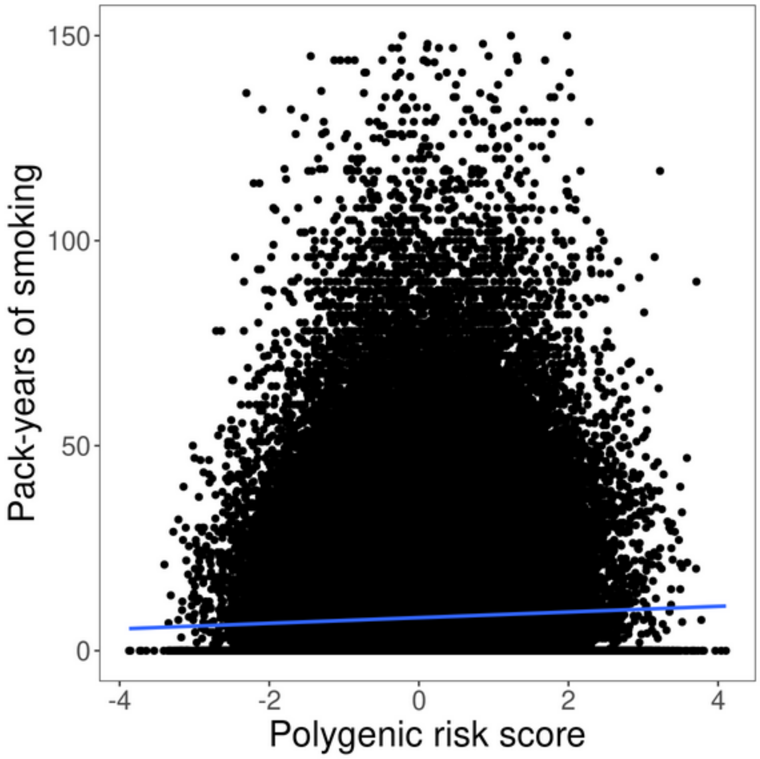

(Pearson $r = 0.041$, $P < .001$).

eFigure 3. The Relationship Between the Polygenic Risk Score and FEV_1/FVC

(A) Ever-smoking vs never-smoking

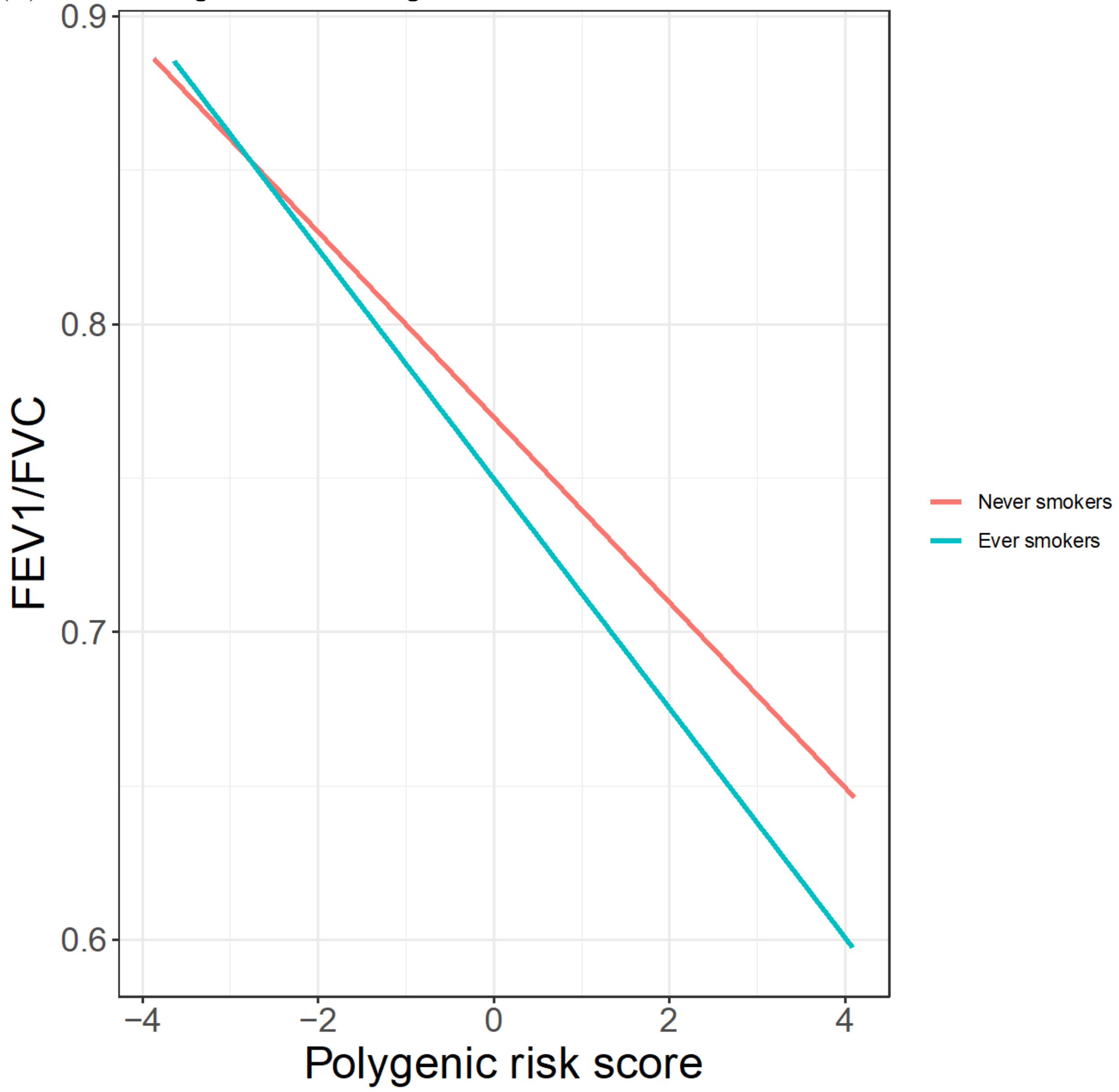

(B) Currently smoking vs formerly or never smoking

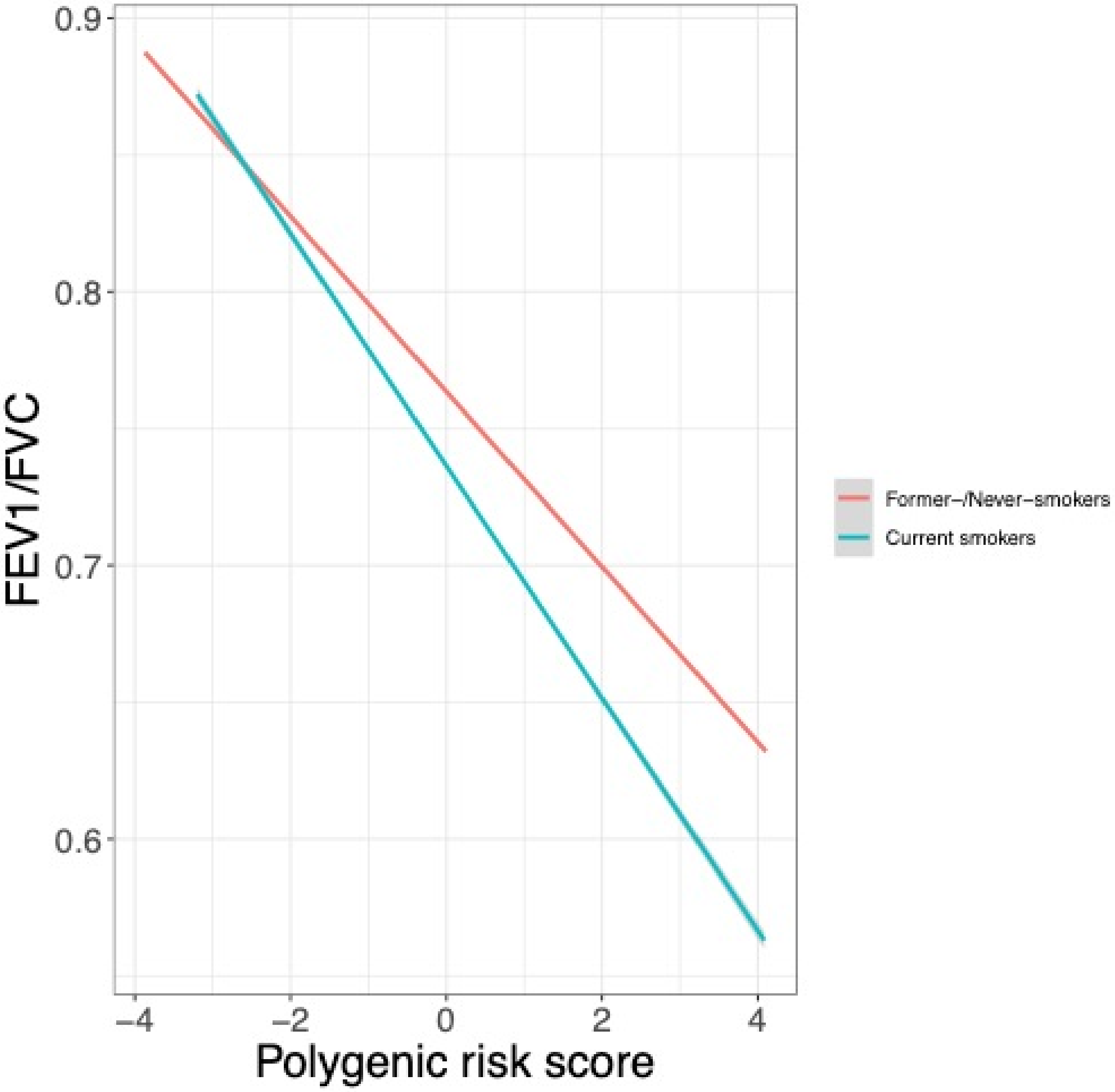

eFigure 4. The relationship between PRS and FEV_1/FVC by pack years of smoking and current vs former smoking status in ever smokers (n=146,679)

A) Pack years of smoking

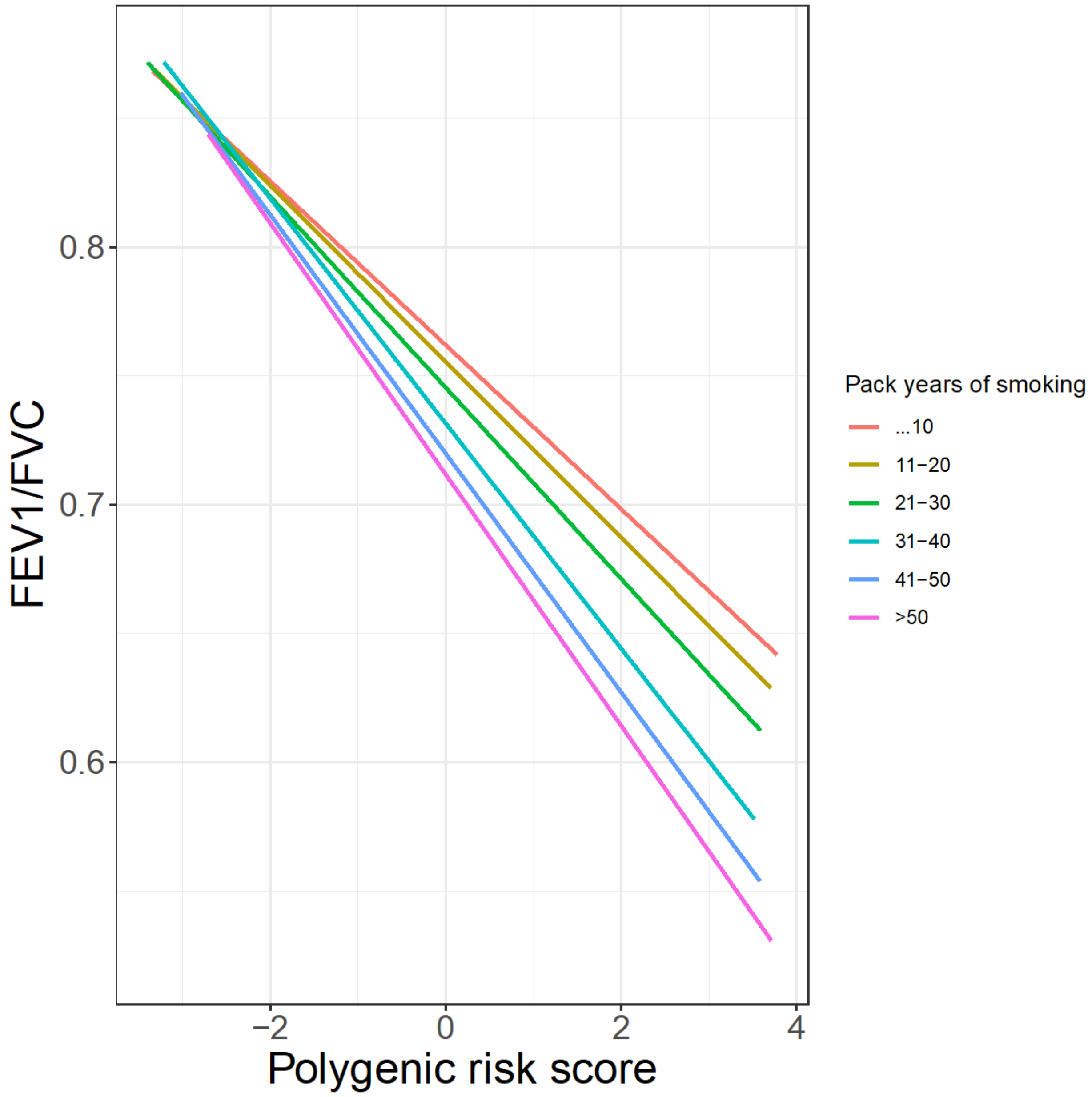

B) Current vs Former Smoking Status

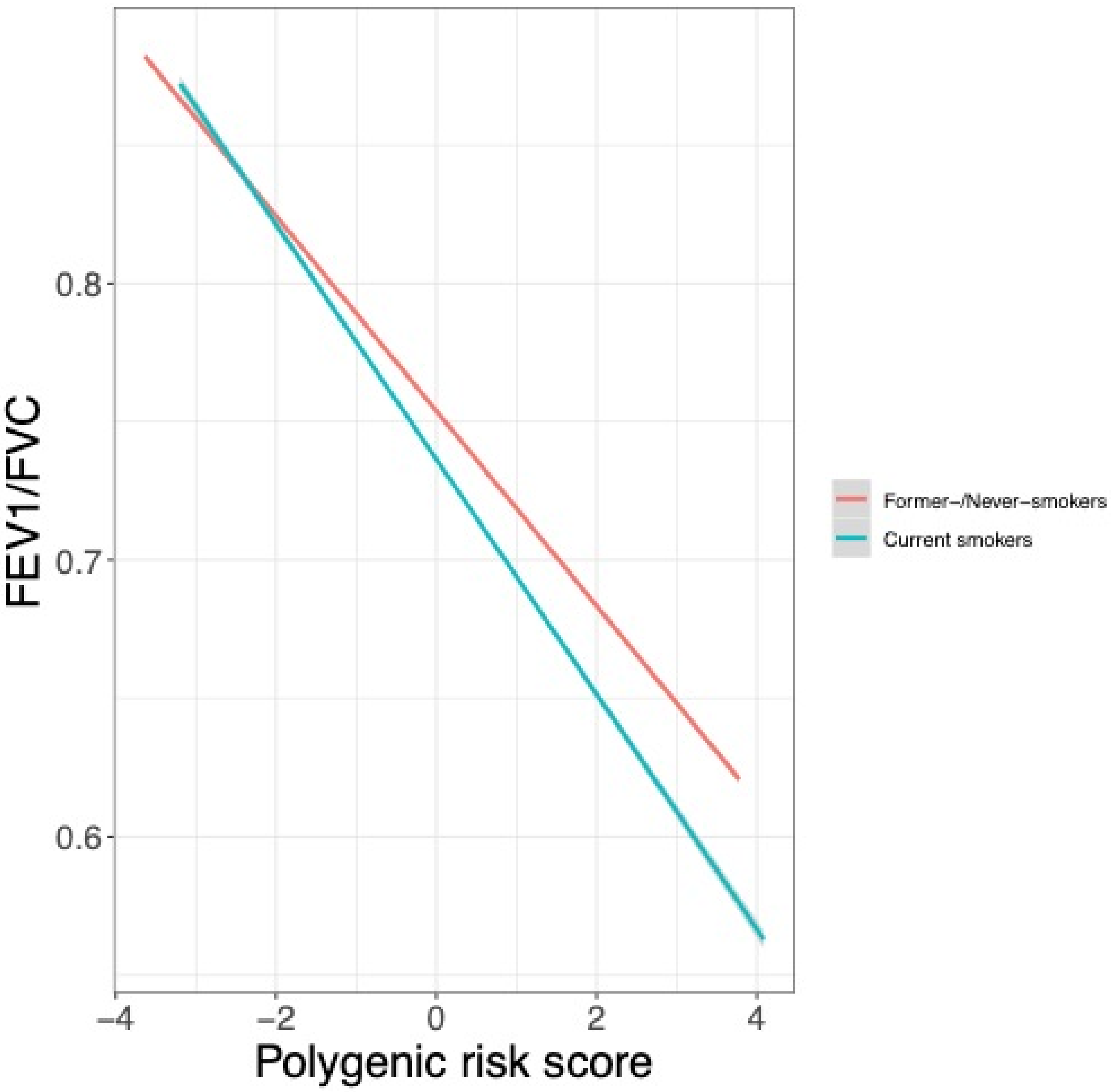

Interaction terms and p-values are shown where appropriate.

© 2021 Kim W et al. *JAMA Network Open.*

eFigure 5. Norms of Reaction for Those With Highest (Tenth) vs Lowest (First) and Middle (Fifth) Genetic Risk for COPD in Individuals Who Had Ever Smoked (n=146,679)

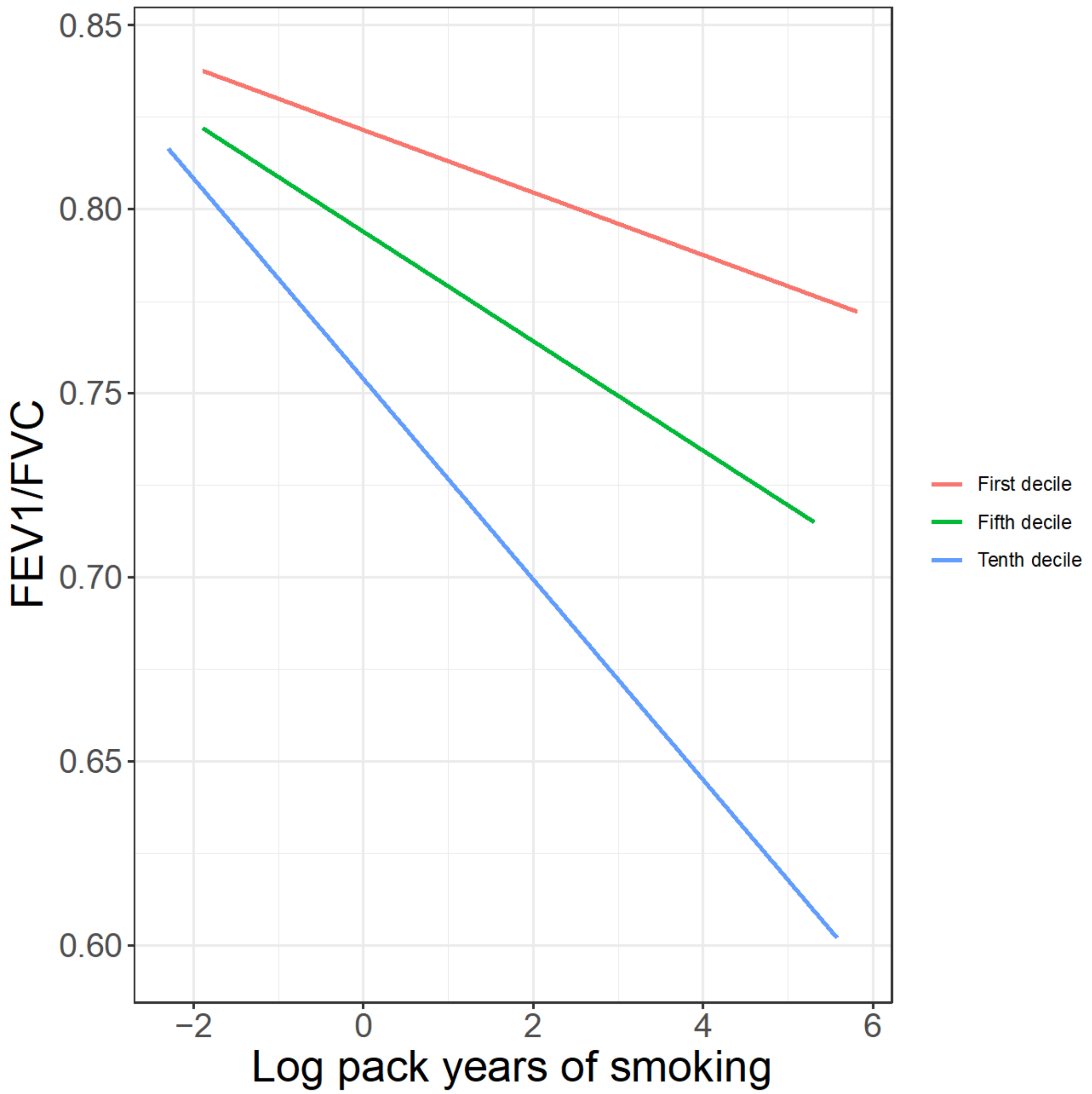

The slopes of the lines for the highest and lowest were significantly different in analysis of covariance ($p < 0.001$).

eReferences

1. Moll M, Sakornsakolpat P, Shrine N, et al. Chronic obstructive pulmonary disease and related phenotypes: polygenic risk scores in population-based and case-control cohorts. *Lancet Respir Med*. 2020;8(7):696-708. doi:10.1016/S2213-2600(20)30101-6
2. Shrine N, Guyatt AL, Erzurumluoglu AM, et al. New genetic signals for lung function highlight pathways and chronic obstructive pulmonary disease associations across multiple ancestries. *Nat Genet*. 2019;51(3):481-493. doi:10.1038/s41588-018-0321-7
